# High Cyclability Energy Storage Device with Optimized Hydroxyethyl Cellulose-Dextran-Based Polymer Electrolytes: Structural, Electrical and Electrochemical Investigations

**DOI:** 10.3390/polym13203602

**Published:** 2021-10-19

**Authors:** Muhammad A. S. Azha, Elham M. A. Dannoun, Shujahadeen B. Aziz, Mohd F. Z. Kadir, Zaki Ismail Zaki, Zeinhom M. El-Bahy, Mazdida Sulaiman, Muaffaq M. Nofal

**Affiliations:** 1 Institute for Advanced Studies, Universiti Malaya, Kuala Lumpur 50603, Malaysia; amirulazha96@gmail.com; 2 Associate Director of General Science Department, Woman Campus, Prince Sultan University, P.O. Box 66833, Riyadh 11586, Saudi Arabia; elhamdannoun1977@gmail.com; 3 Hameed Majid Advanced Polymeric Materials Research Lab, Physics Department, College of Science, University of Sulaimani, Kurdistan Regional Government, Qlyasan Street, Sulaimani 46001, Iraq; shujahadeenaziz@gmail.com; 4 Department of Civil Engineering, College of Engineering, Komar University of Science and Technology, Kurdistan Regional Government, Sulaimani 46001, Iraq; 5 Physics Division, Center for Foundation Studies in Science, Universiti Malaya, Kuala Lumpur 50603, Malaysia; 6 Department of Chemistry, College of Science, Taif University, P.O. Box 11099, Taif 21944, Saudi Arabia; zakimohamed@tu.edu.sa; 7 Department of Chemistry, Faculty of Science, Al-Azhar University, Nasr City, Cairo 11884, Egypt; zeinelbahy@azhar.edu.eg; 8 Chemistry Division, Center for Foundation Studies in Science, Universiti Malaya, Kuala Lumpur 50603, Malaysia; mazdidas@um.edu.my; 9 Department of Mathematics and General Sciences, Prince Sultan University, P.O. Box 66833, Riyadh 11586, Saudi Arabia; muaffaqnofal69@gmail.com

**Keywords:** solid polymer electrolyte, dextran, ammonium bromide, supercapacitors, EDLC

## Abstract

The preparation of a dextran (Dex)-hydroxyethyl cellulose (HEC) blend impregnated with ammonium bromide (NH_4_Br) is done via the solution cast method. The phases due to crystalline and amorphous regions were separated and used to estimate the degree of crystallinity. The most amorphous blend was discovered to be a blend of 40 wt% Dex and 60 wt% HEC. This polymer blend serves as the channel for ions to be conducted and electrodes separator. The conductivity has been optimized at (1.47 ± 0.12) × 10^−4^ S cm^−1^ with 20 wt% NH_4_Br. The EIS plots were fitted with EEC circuits. The DC conductivity against 1000/T follows the Arrhenius model. The highest conducting electrolyte possesses an ionic number density and mobility of 1.58 × 10^21^ cm^−3^ and 6.27 × 10^−7^ V^−1^s^−1^ cm^2^, respectively. The TNM and LSV investigations were carried out on the highest conducting system. A non-Faradic behavior was predicted from the CV pattern. The fabricated electrical double layer capacitor (EDLC) achieved 8000 cycles, with a specific capacitance, internal resistance, energy density, and power density of 31.7 F g^−1^, 80 Ω, 3.18 Wh kg^−1^, and 922.22 W kg^−1^, respectively.

## 1. Introduction

Solid polymer electrolytes (SPEs) have been investigated as electrode separators or ionic conductors in energy source devices (ESDs) by researchers worldwide [1,2]. Due to its exceptional mechanical characteristics, as well as other unique properties, such as being less flammable and having a low toxicity, SPEs have been the subject of active research [3]. Biopolymers offer a successful alternative to non-biodegradable synthetic polymers because of their biocompatibility, biodegradability, and renewability [4]. Common biopolymers that are used in electrolytes are cellulose [5], gelatin [6], chitosan [7], and starch [8]. In recent years, green technologies have become the main focus of the research community due to awareness of the environment. This effort is to reduce the plastic waste in the ocean.

Hydroxyethyl cellulose (HEC) consists of β-1,4 glycosidic linkages that connect the glucose rings [9]. HEC is commonly acknowledged in the cosmetic, paint, and pharmaceutical industries as a thickening and gelling agent. The properties of HEC, such as excellent thermal stability and great electrochemical efficiency, have made it suitable for HEC to be a polymer electrolyte material for many applications [10]. Zhang et al. [11] have documented that two porous polyvinylidene fluoride (PVDF) layers sandwiching a thick HEC membrane has led to an enhancement of electrochemical devices due to its ability in preventing micro short circuits. This is due the hydroxyethyl group attached to HEC’s cellulose backbone. In the study carried out by Li et al. [12], the preparation of a gel membrane is done through soaking the HEC membrane in an organic electrolyte composed of a LiPF_6_ solution in carbonate/ethyl methyl carbonate/dimethyl carbonate, which has shown that the electrolyte could uptake up to 78.3 wt% of the organic liquid electrolyte, also exhibiting excellent electrochemical efficiency and great ionic conductivity at room temperature. HEC can dissolve in different organic solvents, thus making HEC the polymer host for this study [13].

Dextran (Dex) is a natural polymer that is extracted from bacteria (*Leuconostocmesenteroides*) in a sucrose medium. Excess dextransucraseis converted into Dex [14]. There are few studies regarding the employment of Dex as the polymer host although Dex can form a transparent film. Dexpossesses many oxygen functional groups, such as hydroxyl groups (–OH) and glycosidic linkages (C–O–C) [15]. These oxygen atoms contain lone pair electrons that are beneficial for ionic conduction. Moreover, Dex has good water solubility, low toxicity, and relative inertness. Dex has been used in various applications, primarily in medical lines such as plasma expanders, blood substitutes, and bone curing [16].

The desired physical and electrical properties can be enhanced or amplified through the blending of polymers [17]. This method has resulted in polymer blend films that possess better properties than single polymer-based films. Rajendran and Mahendran [18] reported that PMMA-PVA has a higher ionic conductivity than the unblended PMMA and PVA-based electrolytes. A study by Shukur et al. [19,20] has revealed that the ionic conductivity value increased when chitosan is incorporated into a starch–ammonium bromide electrolyte from (5.57 ± 1.88) × 10^−5^ to (9.72 ± 0.95) × 10^−5^ S cm^−1^. Phosphoric acid [21] and sulfuric acid [22] are proton donors but suffer from chemical degradation and deteriorate mechanical integrity, making them less suitable for practical applications [23]. Thus, ammonium salts are regularly reported as proton donors. The most common ammonium salts used are ammonium nitrate (NH_4_NO_3_) [24], ammonium chloride (NH_4_Cl) [25], ammonium thiocyanate (NH_4_SCN) [17], and ammonium bromide (NH_4_Br) [26]. The highest room temperature conductivity achieved by the starch-NH_4_NO_3_ electrolyte is 2.83 × 10^−5^ S cm^−1^ [24]. A report by Hamsan et al. [27] shows that Dex-NH_4_NO_3_ electrolyte obtained a conductivity of 3.00 × 10^−5^ S cm^−1^. A report by Samsudin et al. [28] shows that carboxymethyl cellulose–NH_4_Br exhibited room temperature conductivity at 1.12 × 10^−4^ S cm^−1^.

An electrical double-layer capacitor (EDLC) is one of the alternatives for energy storage device. It can be substituting the conventional chemical batteries as it relies on the energy storage mechanism that obeys the non-Faradaic reactions. Adding salt to the electrolyte produces negatively charged ions (anions) and positively charged ions (cations). Once the EDLC is attached to a power source (charging), one of the electrodes will be positive as the electric field that build around it will attract anions and repel cations. The opposite reaction will occur at the negative electrode. The strong electrical field retains ions from the electrolyte and electrons from the electrode. This is called the formation of a double layer charge where energy is stored as potential energy [29]. Instead, the opposite action takes place during the discharge process. The advantages of EDLC are easy to manufacture, safe, and cheap. Moreover, EDLCs have a longer life cycle, higher power density, more excellent reversibility, and better thermal stability, unlike Faradaic capacitors or pseudo capacitors [30]. This study reports the electrical behavior of Dex-HEC blend-based electrolytes incorporated with NH_4_Br. Ammonium salt was chosen as it is reported to be suitable for low-energy-density device applications. The highest conducting electrolyte was used in EDLC as the separator of electrodes.

## 2. Experimental

### 2.1. Electrolyte Preparation

In 100 mL of distilled water, different amounts of HEC (Sigma Aldrich, Kuala Lumpur, Malaysia) were dissolved. Dex (Sigma Aldrich, avgmolwt 35,000–45,000) was then added after the HEC solution had been fully dissolved. The mixture was stirred until the solution became homogeneous. The most amorphous system was selected to prepare the solid-based electrolytes. Various NH_4_Br (Bendozen, Kuala Lumpur, Malaysia) quantities were employed to prepare the salted system in the most amorphous Dex-HEC solutions, and the mixture was stirred until complete dissolution was achieved. The solutions were cast into different Petri dishes and allowed to dry for 4–5 days (25 °C, relative humidity, RH ~ 50%). All the films were stored in a silica geldesiccator package and then coded as presented in Table 1 and Table 2.

### 2.2. Electrolyte Characterization

For the XRD analysis, a Siemens D5000 X-ray diffractometer (1.5406 Å) (Malvern Panalytical Ltd., Malvern, UK) was used. The angle of 2*θ* varied from 5° to 80° with a resolution of 0.1°. The degree of electrolyte crystallinity (*χ_c_*) was checked with

(1) $$\chi_{c}=\frac{A_{cry}}{A_{s}}\times 100\%$$

where the areas are defined as *A_s_* and *A_cry_*, for the sum of the hump and crystalline peaks, respectively. The deconvolution method (OriginPro8 Software) was used to calculate the areas of the peaks.

The impedance study was performed using the frequency range between 50 Hz and 5 MHz using a HIOKI 3532-50 LCR HiTESTER (HIOKI, Nagano, Japan) at room temperature. An AC voltage of the peak-to-peak amplitude of 10 mV was superimposed onto the DC voltage of 40 mV. The electrolytes were sandwiched between two electrodes made of stainless steel. The electrolyte’s conductivity was determined using the following formula:

(2) $$\sigma =d\times R_{b}^{-1}A^{-1}$$

where *R_b_* denotes the bulk resistance, *A* stands for the electrolyte-electrode contact surface area, and *d* is the electrolyte’s thickness. The results of the impedance analysis were also employed in the dielectric study analysis. The stability of the electrolyte against the applied potential was measured using linear sweep voltammetry (LSV) analysis at a sweep rate of 5 mV s^−1^. Cyclic voltammetry (CV) was also performed to show the capacitive behavior of the sample at various scan rates. The cell was connected to the three electrode systems, namely, the counter electrode, working electrode, and reference electrode, using a Digi-IVY DY2300 potentiostat. The current was measured between the working electrode and the counter electrode while the potential was measured between the reference electrode and the working electrode.

### 2.3. EDLC Preparation

The EDLC was constructed using carbon black, PVDF, and activated carbon. Under gentle stirring, 0.50 g of PVDF was incorporated into 15 mL of N-methyl pyrrolidone (NMP). Using a planetary ball miller, a mixed powder was created by mixing 0.25 g of black carbon with 3.25 g of activated carbon. After that, the mixed powder was added to the NMP-PVDF solution. The mixture was then cast on an aluminum foil with a doctor blade and heated for a certain amount of time at 60 °C. To eliminate excess moisture, the electrodes were placed in a desiccator containing silica gel. An area of 2.01 cm^2^ was taken out of the electrode. The highest conductive electrolyte was sandwiched between two electrodes, then packed into a CR2032 coin cell. The electrode had a thickness of 0.0025 cm. At a potential range of 0 to 0.9V and a sweep rate of 5, 10, 20, 50, and 100 mV s^−1^, the Digi-IVY DY2300 potentiostat was utilized to conduct cyclic voltammetry (CV) analysis.

## 3. Result and Discussion

### 3.1. Ideal Composition for the Polymer Blend Host of the Electrodes Separator

**Figure 1:**
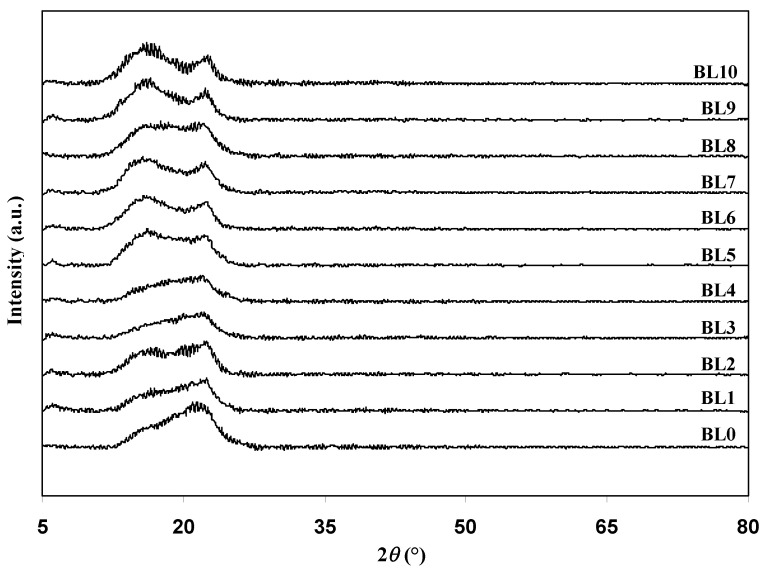
XRD patterns for the polymer blend.

Figure 1 shows the XRD patterns of the polymer blends. The pattern of the XRD for BL0 has two humps at 2*θ* = 15.8° and 22.64°, where the peak at 15.8° is less prominent. This is a typical XRD pattern of semi-crystalline materials. As more Dex is added into the polymer blend, these two peaks changed their intensity and position. The trend inthe XRD pattern moved towards the pattern of BL10 as more Dex was added. This indicates that the oxygen-containing functional groups of Dex and hydrogen in the structure of HEC, or vice versa, have formed an interaction via hydrogen bonding. Furthermore, it is a sign that the crystalline structure in the blend has been reduced [31]. From the plot in Figure 1, BL4 has the most negligible intensity among other blends, which signifies that BL4 has the most amorphous structure compared to the other blends. However, this statement has to be further confirmed through the deconvolution technique as it helps to separate any potential amorphous and crystalline peaks that may overlap. In this method, prominent peaks signify amorphous regions, while the narrow peaks indicate that the region is crystalline [32].

**Figure 2:**
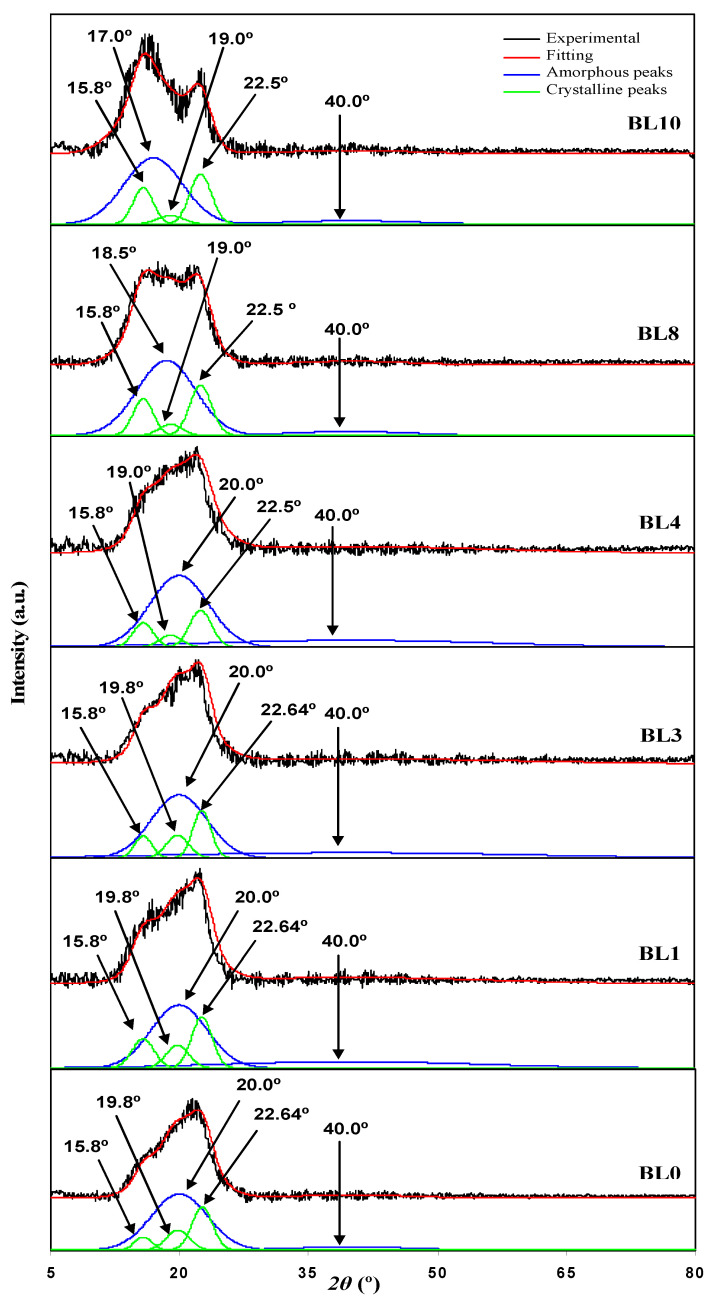
Deconvoluted XRD patterns for the polymer blend.

Figure 2 shows the deconvoluted XRD patterns of the chosen polymer blend films. BL0 and BL10 can serve as the references for detecting changes in the peak position or intensity in the diffractogram of the polymer blends. The XRD pattern of BL0 has three crystalline peaks at 2*θ* = 15.8°, 19.8°, and 22.64°, as well as two amorphous peaks at 2*θ* = 20.0° and 40.0°. This finding is similar to the results obtained by Hanif et al. [33]. In another study [34], for BL10, three crystalline peaks at 2*θ* = 15.8°, 19.0°, and 22.5° and two amorphous peaks at 2*θ* = 20.0° and 40.0° have similarly appeared [34]. The XRD pattern in BL4 indicates a reduction in the intensity of three crystalline peaks at 2*θ* = 15.8°, 19.0°, and 22.5°, indicating the increase in amorphousness of the blend. Equation (1) calculates the degree *χ*_c,_ and the results are tabulated in Table 3. BL4 blend has the most amorphous blend as it displays the lowest *χ*_c_; therefore, the blend of 40 wt% Dex and 60 wt% HEC is chosen as the polymer host.

### 3.2. Conductivity Analysis

**Figure 3:**
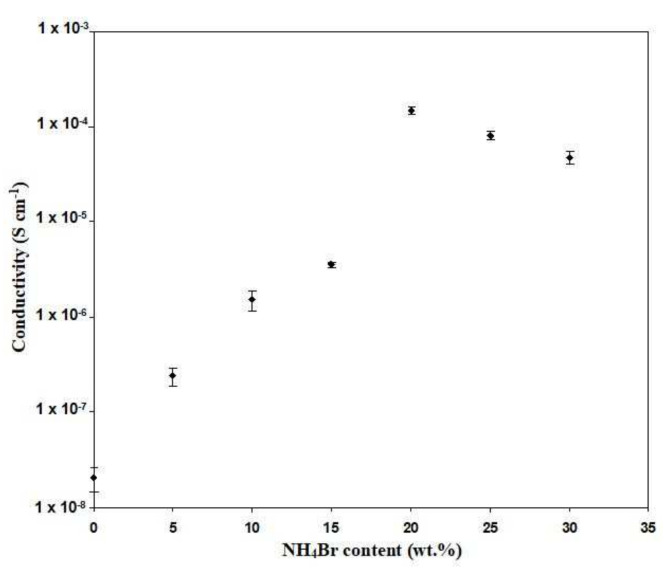
Conductivity versus NH_4_Br content at room temperature.

**Figure 4:**
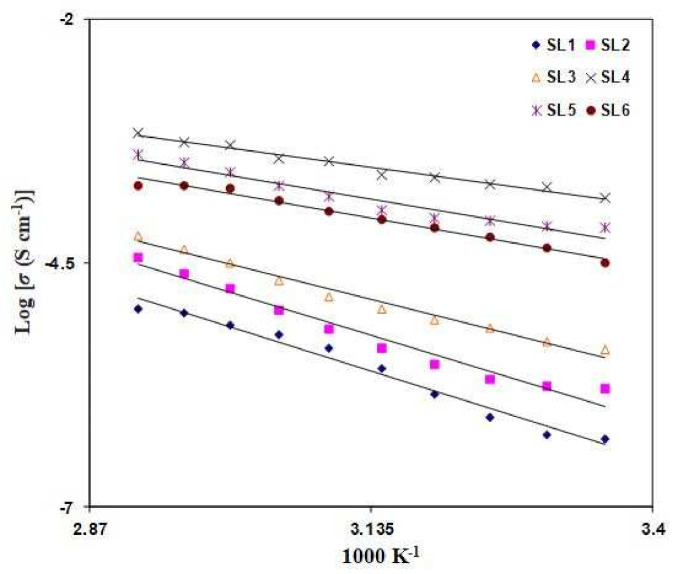
Conductivity at various temperatures for the salted electrolytes.

Figure 3 displays the variation in room temperature DC conductivity (with error bars) when the NH_4_Br content is changed. It is observed that the conductivity increases from (2.04 ± 0.57) × 10^−8^ to (2.42 ± 0.54) × 10^−7^ S cm^−1^ as 5 wt% of NH_4_Br is added into the Dex-HEC host. It can be seen that the conductivity value has been maximized up to (1.47 ± 0.12) × 10^−4^ S cm^−1^ with the inclusion of 20 wt% NH_4_Br into the polymer host. This inclination in the conductivity value is a result of the increment in the charge carrier [24]. However, the trend stops at 20 wt% NH_4_Br content. Any increment surpassing the 20 wt% of NH_4_Br content leads to a decreasing conductivity value. This finding was verified by Mohamed et al. [35], who concluded that the reduction in conductivity value is due to ion pairs, ion triplets, and higher ion aggregations, which significantly reduce the mobility and ionic number density. The variation in conductivity for the salted electrolytes as a function of temperature is depicted in Figure 4. Conductivity is observed to increase with the rise of the temperature. Due to the linear relation between conductivity and temperature (R^2^ ~ 0.97–0.99), all electrolytes in this study obey the Arrhenius law. Therefore, the polymer host structure does not have a phase transition when the salt is added [36]. The equation of Arrhenius is given as

(3) $$\sigma =\sigma_{o}\exp\left(-\frac{E_{ac}}{kT}\right)$$

where *E_ac_* is the ion activation energy for the migration from one site to another, *k* is the Boltzmann constants, $\sigma_{o}$ stands for a pre-exponential component, and *T* is the absolute temperature. Other proton-based polymer electrolyte studies have also reported Arrhenius behavior, such as PVA-NH_4_SCN [37], MC-PVA-NH_4_NO_3_ [38], and PVA-NH_4_Cl [23]. The plot slope in Figure 4 is used to evaluate *E_ac_*. As illustrated in Table 4, the *E_ac_* value is 0.68 when 5 wt% NH_4_Br is infused and further decreases to 0.26 with the presence of 20 wt% NH_4_Br. Sohaimy et al. [39] reported that the highest conducting electrolyte, carboxymethylcellulose (CMC) doped with ammonium carbonate ((NH_4_)_2_CO_3_), has the lowest *E_ac_* value. The number density of ions in the electrolyte increases as the NH_4_Br concentration rises, reducing the energy barrier [40]. As a result, the increased conductivity value is explained. In the transport section, the rise in conductivity with rising temperature will be discussed further.

### 3.3. Impedance Analysis

**Figure 5:**
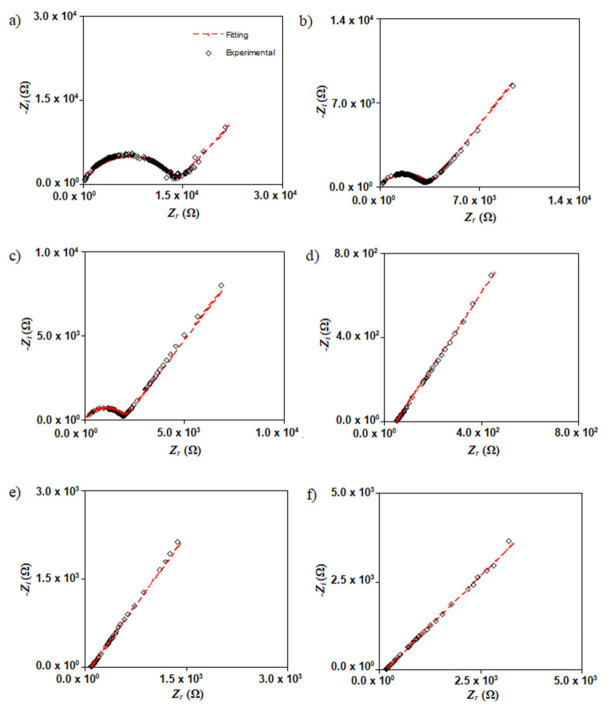
Nyquist plot for (**a**) SL1, (**b**) SL2, (**c**) SL3, (**d**) SL4, (**e**) SL5 and (**f**) SL6 at room temperature.

**Figure 6:**
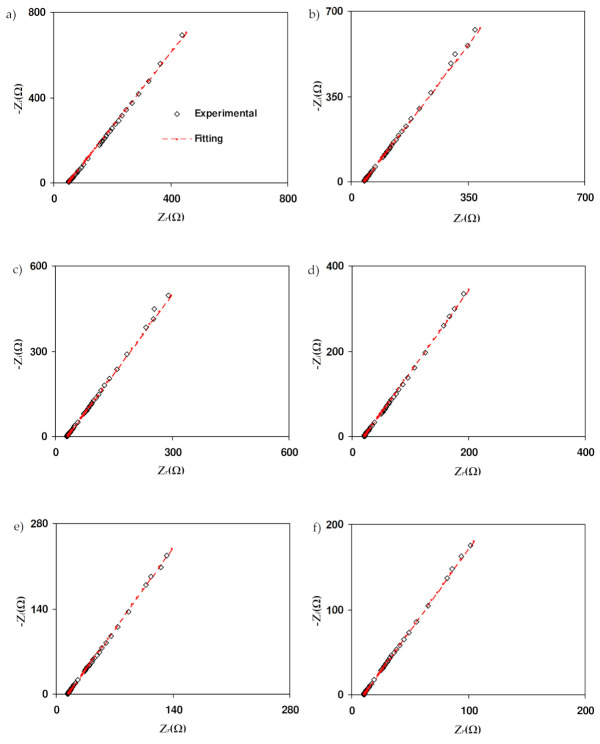
Nyquist plot for SL4 at (**a**) 298K, (**b**) 303K, (**c**) 313K, (**d**) 323K, (**e**) 333K and (**f**) 343K.

Figure 5 displays the Nyquist plot for SL1, SL2, SL3, SL4, SL5, and SL6 at room temperature. SL1 (Figure 5a) shows a semicircle and spike. *R_b_* can be obtained from the interception of the semicircle and spike. Ionic migration in the bulk of electrolytes caused the shape of the semicircle at higher frequencies while the spike at low frequencies indicates a polarization effect [41]. As the concentration of salt increases, theSL2 and SL3 semicircles decreased; this denotes that there are more ions available. Thus, more ions can travel from one electrode to the other, as the concentration of NH_4_Br increases [42]. As seen in Figure 5d, the addition of 20 wt% NH_4_Br (SL4) resulted in the disappearance of the semicircle. Compared with the previous SL1, *R_b_* is taken from the intersection point of the spike and horizontal axis. Shukur [43] stated that the disappearance of the semicircle is due to the dominance of resistive parts of the polymer. This condition also suggests that there is an increment in the number density of ions. The *R_b_* value for SL5 and SL6 increase, implying that the salt was recrystallized [44]. This means that the conductivity value of SL5 and SL6 is lower than SL4. The SL4 electrolyte is selected for impedance analysis at different temperatures, as shown in Figure 6. The increment in temperature leads to a decrease in the *R_b_* value. The temperature enhances the salt dissociation and the segmental movement of the polymer as well [45]. Hence, the electrolyte conductivity will be increased as the temperature rises.

**Figure 7:**
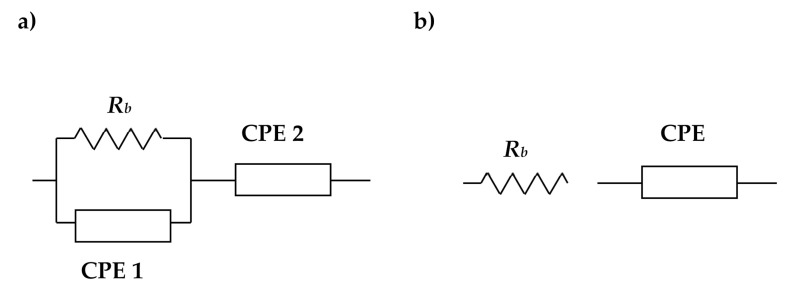
Equivalent circuit for (**a**) the semicircle-spike plot and (**b**) spike plot.

The equivalent electrical circuit is used because it gives an easy and direct picture of the device. Figure 7a,b shows the corresponding circuit for the semicircle-spike and spike plots, respectively. Due to the electrolyte’s inhomogeneity, it was categorized as an “imperfect capacitor”, known as the constant-phase element (CPE). CPE is given as [46]

(4) $$Z_{CPE}=\frac{1}{C\omega^{p}}\left[\cos\left(\frac{\pi p}{2}\right)-i\sin\left(\frac{\pi p}{2}\right)\right]$$

where *p* relates to the plot’s deviation from the axis. *Z_i_* and *Z_r_* are the imaginary and real parts of the impedance, respectively. *C* is the CPE’s capacitance, while *ω* is the radial frequency. The circuit in Figure 7a (for the spike and semicircle) can be expressed via the following equations:

(5) $$Z_{r}=\frac{R_{b}C_{1}\omega^{p1}\cos\left(\frac{\pi p_{1}}{2}\right)+R_{b}}{2R_{b}C_{1}\omega^{p}\cos\left(\frac{\pi p}{2}\right)+R_{b}{}^{2}C_{}{}^{2}\omega^{2p}+1}+\frac{\cos\left(\frac{\pi p_{2}}{2}\right)}{C_{2}\omega^{p2}}$$

(6) $$Z_{i}=\frac{R_{b}C_{1}\omega^{p1}\sin\left(\frac{\pi p_{1}}{2}\right)}{2R_{b}C_{1}\omega^{p}\cos\left(\frac{\pi p}{2}\right)+R_{b}{}^{2}C_{}{}^{2}\omega^{2p}+1}+\frac{\sin\left(\frac{\pi p_{2}}{2}\right)}{C_{2}\omega^{p2}}$$

where *p*_2_ is the spike deviation from the horizontal axis and *p*_1_ is the spike deviation from the vertical axis for semicircle deviation. *C*_1_ and *C*_2_ are related to the high- and low-frequency capacitance, respectively. As shown in Figure 7b, the impedance plot with a spike has a circuit of series combination between *R_b_* and CPE. This set of impedance can be expressed in these equations:

(7) $$Z_{r}=\frac{\cos\left(\frac{\pi p}{2}\right)}{C\omega^{p}}+R_{b}$$

(8) $$Z_{i}=\frac{\sin\left(\frac{\pi p}{2}\right)}{C\omega^{p}}$$

The circuit elements for salted electrolytes with various circuit element parameters have been determined and are shown in Table 5 and Table 6. Based on Table 5, the *C* value is higher at a low frequency than at a high frequency. This phenomenon is consistent with the following equation:

(9) $$C=\frac{A\varepsilon_{o}\varepsilon_{r}}{d}$$

In this equation, *ε_r_* and *ε_o_* stand for the dielectric constant and vacuum permittivity, respectively. The value of *ε_r_* is low at a high frequency, which, successively, gives a low value of *C* [47]. Dielectric information will be further explained in the dielectric section. It is observed that the value of *C* increases as more salt is incorporated. The high value of *C* indicates that more ions are produced at a high concentration of salt. Table 6 shows the circuit element of SL4 at 298, 303, 313, 323, 333, and 343 K, respectively. The increase in *C* at a higher temperature is due to the enhancement of mobile ions at a high temperature. Shuhaimi et al. [48] stated that the rise in *C* value with increasing temperature means that the electrolyte is suitable for the implementation in technologies of electrochemical devices.

### 3.4. Determination of Transport Parameters

Transport analysis is a pivotal study as it identifies the value of the mobility (*μ*) of ions and the number density (*n*). The conductivity value is affected by these two parameters, *n* and *μ*, as shown in the following equation:

(10) σ=neμ

where *e* is the electronic charge. The Arof-Noor (A-N) method is used to study the transport properties of electrolytes [49]. By utilizing the A-N method, it enables us to determine the transport properties of impedance analysis. The *μ* value is expressed as:

(11) μ=eDkT

where the coefficient of diffusion (*D*) for impedance plots with a combination of a semicircle and spike can be described as

(12) D=Ak2εrεo2τ2

**Figure 8:**
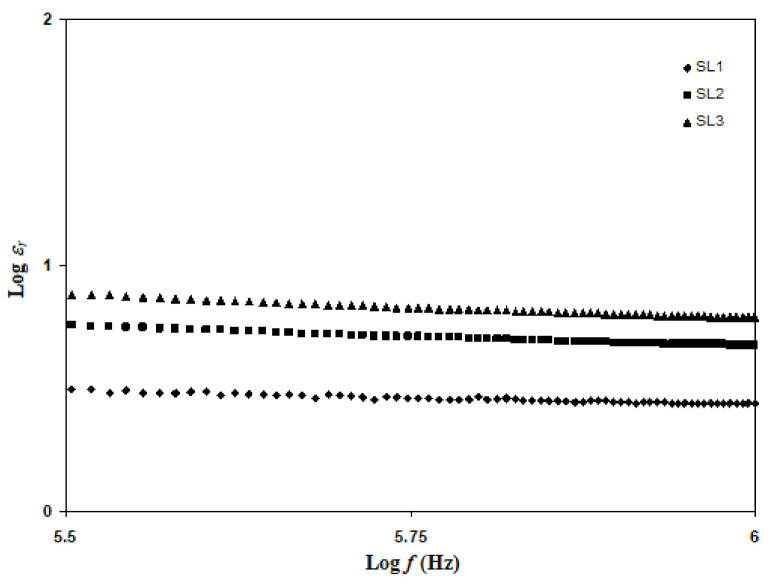
Log *ε_r_* vs. log *f* for SL1, SL2, and SL3.

Here, *k*_2_ can be extracted from the impedance where *k*_2_ is inverse to *C*_2_, and *τ*_2_ is taken from the *Z_i_* minimum. Fadzallah et al. [50] stated that the value of *ε_r_* can be determined where log *ε_r_* is constant. For impedance plots with a spike only, the value of *D* can be determined from

(13) $$D=D_{o}\exp\left\{-0.0297{\left[\ln D_{o}\right]}^{2}-1.4348\left[\ln D_{o}\right]-14.504\right\}$$

(14) $$D_{o}=\left(4k^{4}d^{2}\right)\times {\left(R_{b}{}^{4}\omega_{m}^{3}\right)}^{-1}$$

where *ω_m_* stands for angular frequency associated with the minimum *Z_i_*, while *k* is the inverse of *C* of the impedance fitting. In Figure 5, the Nyquist plot of SL1, SL2, and SL3 consists of a semi-circle and an inclined spike. Equation (12) is therefore used to calculate the value of *D*. According to Equation (12), by plotting the log *ε_r_* vs. log *f*, the constant value of *ε_r_* can be determined. Based on Figure 8, *ε_r_* is obtained by log *ε_r_* > 5.5.

**Figure 9:**
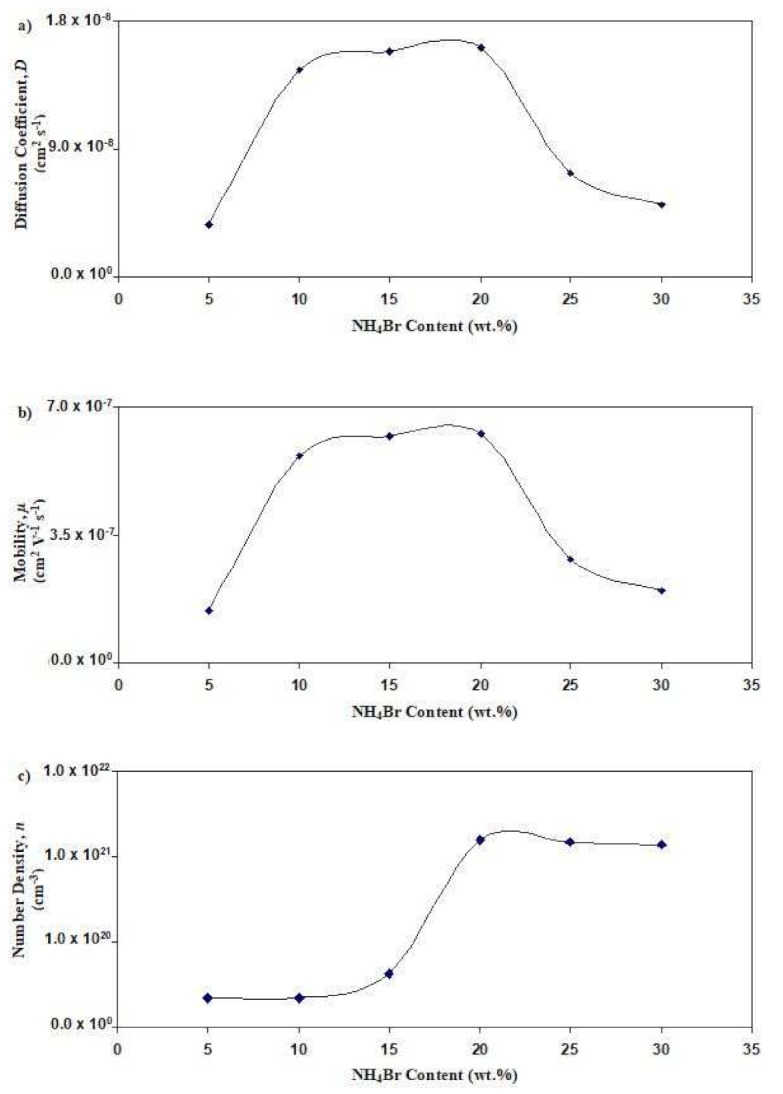
Graph of (**a**) *D*, (**b**) *μ*, and (**c**) *n* for the salted electrolytes at room temperature.

**Figure 10:**
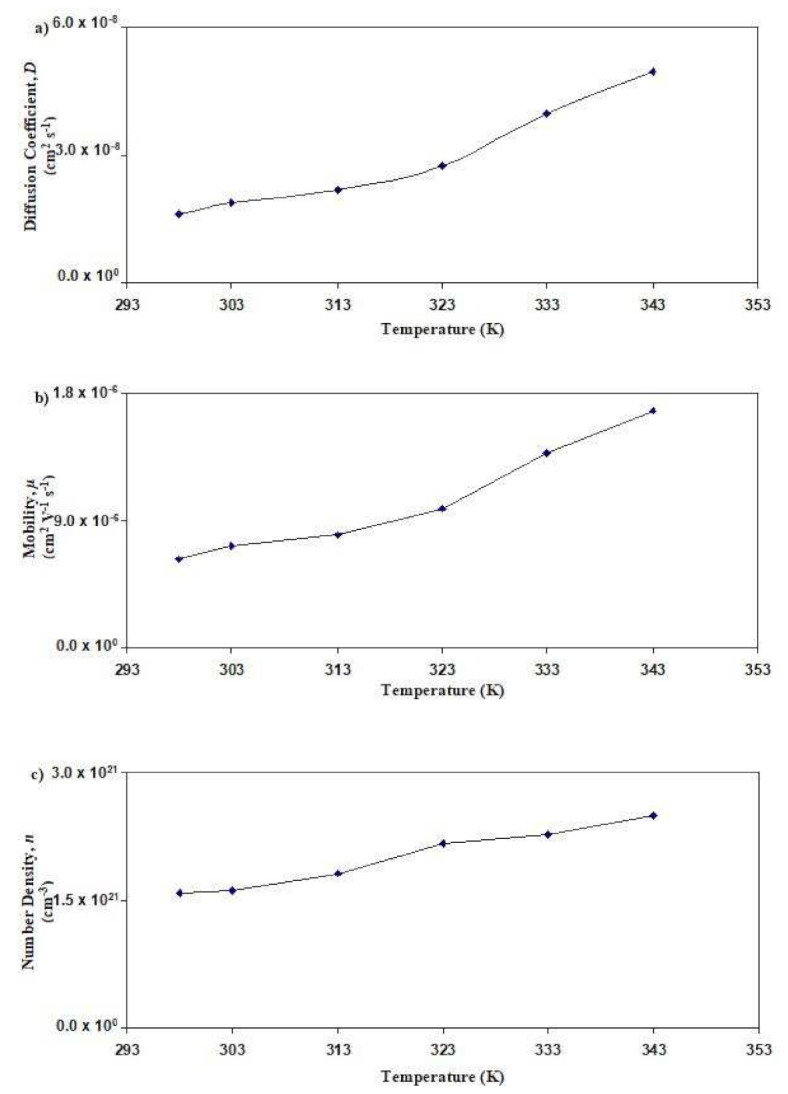
Graph of (**a**) *D*, (**b**) *μ*, and (**c**) *n* for the SL4 electrolytes at various temperatures.

Figure 9 displays the salted electrolyte’s transport parameters at room temperature. In Figure 9a, the concentration of NH_4_Br increases from 5 wt% to 20 wt%; it is observed that the value of *D* increases from 3.65 × 10^−9^ cm^2^ s^−1^ to 1.61 × 10^−8^ cm^2^ s^−1^, respectively. However, when 25 wt% and 30 wt% of NH_4_Brare added, the value of *D* drops to 7.26 × 10^−9^ cm^2^ s^−1^ and 5.02 × 10^−9^ cm^2^ s^−1^, respectively. A similar pattern is observed for *μ* as displayed in Figure 9b, as the most conductive salted electrolyte (with 20 wt% NH_4_Br) has the highest value of *μ* (6.27 × 10^−7^ cm^2^ V^−1^ s^−1^). In Figure 9c, it is observed that the value of *n* at 20 wt% NH_4_Br achieved the maximum value of 1.58 × 10^21^ cm^−3^. The *n* value decreases as the concentration of NH_4_Br reaches 25 wt%. This shows that the ionic conductivity trend at room temperature for the salted electrolyte is aligned with the trend of *D*, *μ*, and *n*. Hence, it can be concluded that the decrement of the transport parameters at a high salt content is relatively caused by the close distance between the cations and anions, which leads to the reduction in the number density of the ion and ionic conductivity [51]. Figure 10 displays the transport parameters for SL4 at various temperatures. Figure 10a shows that the *D* value increases from 1.61 × 10^−8^ cm^2^ s^−1^ to 4.97 × 10^−8^ cm^2^ s^−1^ as the temperature increases from 298 K to 343 K. The same pattern for *μ* is obtained, whose maximum value is observed in Figure 10b at 1.68 × 10^−6^ cm^2^ V^−1^ s^−1^. It is visible from Figure 10c that the value of *n* increases from 1.58 × 10^21^ cm^−3^ to 2.51 × 10^21^ cm^−3^. This segment of polymers vibrates at high temperatures, resulting in free volume enhancement. This volume allows ion pairs to dissociate and form free ions that indirectly increase the diffusivity and mobility of ions.

### 3.5. Linear Sweep Voltammetry (LSV)

**Figure 11:**
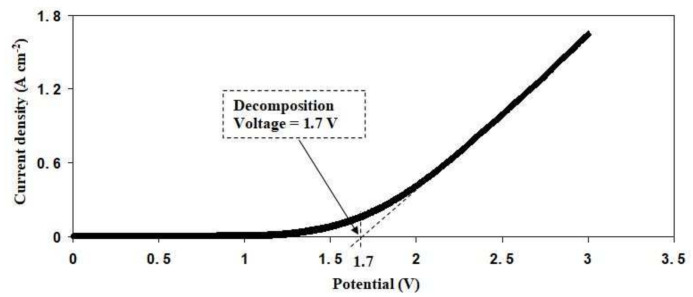
LSV curve for the SL4 electrolyte.

A linear sweep voltammetry (LSV) study is an essential tool used to check the suitability of the electrolyte in electrochemical device applications. Figure 11 displays the LSV plot for SL4. There are no apparent changes in the current value at a potential less than 1.7 V as the potentials are swept linearly from 0 to 3 V. This means that SL4 is electrochemically stable up to 1.7 V [52]. The increase in current over 1.7 V is due to the electrolyte decomposition at the surface of the inert electrode [53]. Aziz et al. [54] reported an ammonium salt-based biopolymer electrolyte, where it started to decompose at 1.27 V. This finding is very similar to other polymer electrolytes with an ammonium salt. Electrochemical stability up to 1.6 V has been reported by Noor and Isa [55] for a polymer electrolyte system of CMC doped with NH_4_SCN. The CS-NH_4_I-Zn-glycerol system is reported to be potentially stable up to 1.37 V [53]. As a consequence of this finding, it can be stated that the highest conducting electrolyte provides sufficient potential stability for the use of energy storage devices.

### 3.6. Cyclic Voltammetry (CV)

**Figure 12:**
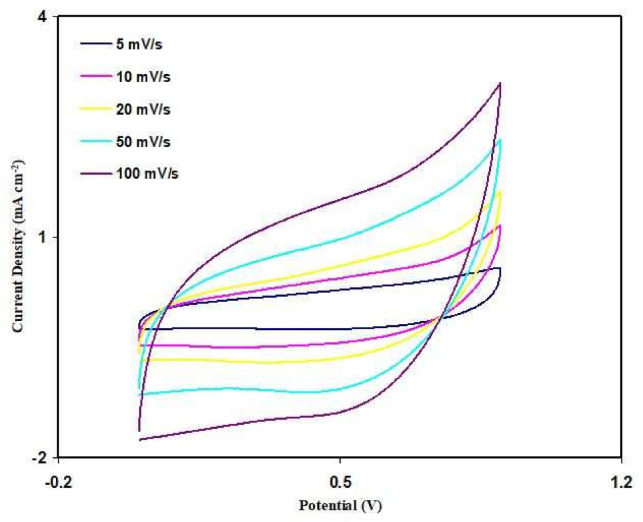
Cyclic voltammetry (CV) curve of the developed electrical double-layer capacitor (EDLC) for the SL4 film.

Cyclic voltammetry (CV) analysis has been used to test the EDLC fabricated using SL4. The CV curve for the fabricated EDLC is shown in Figure 12 at a series of scan rates. It is observed that redox reactions do not occur in the potential range between 0 and 0.9 V, as the peaks that indicate that situation is absent. This situation tallies with the energy storage mechanism of an EDLC [56]. It is noticeable that as the scan rate decreases, the CV shape shifts from a leaf shape to nearly a rectangular shape. Kant et al. [57] mentioned that the rectangular CV curve indicates a good charge transport. The specific capacitance (*C_CV_*) of the EDLC can be obtained from the CV curve using the following equation:

(15) $$C_{CV}=\int_{V_{i}}^{V_{f}}\frac{I(V)dV}{2ma(V_{f}-V_{i})}$$

where the area of the CV curve (∫*I*(*V*)*dV*)in cm^2^ is extracted using the integration feature of Origin 8.5 software. The scan rate in V/s is represented by *a*, while the mass of the activated material with gram (g) is represented by *m*. V_f_, and V_i_, respectively, representing the final voltage of 0.9 V and the initial voltage of 0 V. Table 7 shows the calculated values of *C_CV_*. As the scan rate decreases, the *C_CV_* value increases. Ions can form proper Helmholtz layers at the surface of activated carbon electrodes at low scan rates. At higher scan rates, some ions undergo recombination and the transportation of ions is unstable, thus providing a low capacitance value [58,59].

### 3.7. Dex-HEC-NH_4_Br-Based EDLC

**Figure 13:**
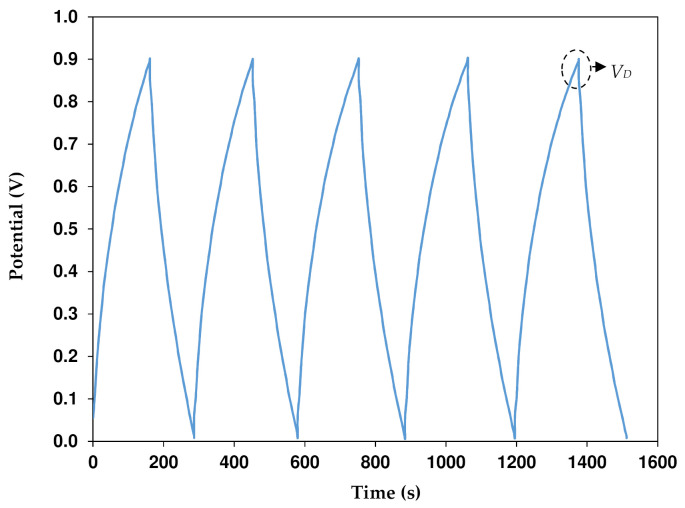
Charge-discharge profiles for the EDLC at 0.25 mA cm^−2^.

**Figure 14:**
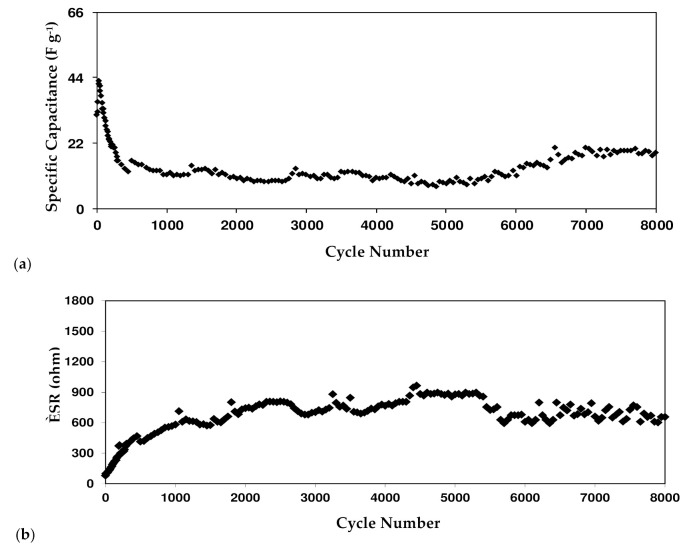

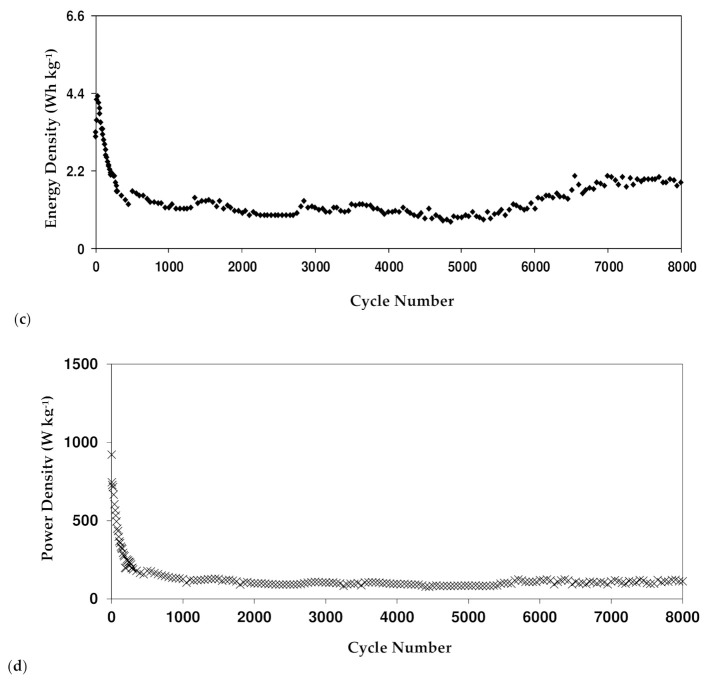
EDLC parameters: (**a**) specific capacitance, (**b**) equivalent series resistance, (**c**) energy density, and (**d**) power density of the EDLC for 8000 cycles.

At 0.25 mA cm^−2^ and a potential range of 0 to 0.9 V, the EDLC’s rechargeability is tested for 8000 cycles. Figure 13 shows the EDLC’s charge-discharge plot. The EDLC’s capacitive behavior can be seen by the discharge slope, which is nearly linear [60]. Since the slope of the discharge curve (s) is known, the specific capacitance (*C_CD_*) can be calculated using the following equation:

(16) $$C_{CD}=\frac{i}{sm}$$

where *i* is the constant current. Figure 14a shows the *C_CD_* of the EDLC where the 1st cycle is 31.7 F g^−1^. This value is comparable to the one obtained from CV analysis. The *C_CD_* increases to 42.8 F g^−1^ as the cycle number reaches the 30th cycle but reduces to 12.4 F g^−1^ until 450th cycles. Some ions are recombined in the rapid charge and discharge process creating ion pairs or aggregations. Ion pairs or aggregations block the conduction of ions, thus reducing the value of the capacitance [61]. It is common in EDLC that at the beginning cycles the performance is unstable as cations and anions are still trying to recognize the polarization pattern. The stabilization of *C_CD_* can be observed from 450th to the 8000th cycle with an average of 13.1 F g^−1^. Table 8 shows EDLC reported in other works with their respective parameters.

The ideal charge-discharge curve is similar to a triangular shape. However, the poor triangular shape could be due to the roughness of the carbon electrode, the type of electrolytes, and the internal resistance. As shown in Figure 13, the discharge process is started by a slight potential drop (*V_D_*). It can be linked to internal resistance in the EDLC, which is called the equivalent series resistance (*R_ESR_*). Based on the following equation, we can obtain the *R_ESR_*:

(17) $$R_{ESR}=\frac{V_{D}}{i}$$

Figure 14b shows the *R_ESR_* of the EDLC for 8000 cycles and it varies from 80 to 880 Ω. To begin, a double-layer charge or potential energy might emerge in the gap between the electrolyte and the electrode. The presence of ions from the electrolyte and electrons from the carbon electrode results in a double-layer charge [62]. Second, it is from the electrolyte itself that the fast charge/discharge process induces recombination of free ions and reduces the ionic conductivity. Finally, it is the current collectors. In this case, it is aluminum foil.

The energy density (*E_D_*) of the EDLC can be expressed as

(18) $$E_{D}=\frac{C_{CD}V}{2}$$

where *V* is the applied voltage (0.9 V). *E_D_* is found to be 3.18 Wh kg^−1^ in the 1st cycle in Figure 14c. The stabilization can be observed as soon as the cycle number reaches 450 where it fluctuates with an average of 1.31 Wh kg^−1^ up to the 8000th cycle. The pattern of *E_D_* is harmonized with the pattern of *C_CD_*. This stabilization of the *E_D_* values indicates that the ions face the same energy barrier during migration towards the electrodes from 450th to 8000th cycle. [32] Another crucial parameter of the EDLC is power density (*P_D_*) which can be determined using:

(19) $$P_{D}=\frac{V^{2}}{4mR_{ESR}}$$

As shown in Figure 14d, the value of *P_D_* is plotted up to 8000 cycles. In the 1st cycle, the value of *P_D_* is 922.22 W kg^−1^, but it decreases down to 319.6 W kg^−1^ at the 150th cycle. The *P_D_* then further reduces to 158.4 W kg^−1^ at the 450th cycle before it become constant at an average of 109 W kg^−1^. This may be linked to the electrolyte depletion and growth of ion aggregates/pairs during the quick charge-discharge process. Ion aggregates/pairs are the critical drawbacks of polymer electrolyte membranes. As a result, the accumulated ion in the double-layer formed at the surface of the electrodes is lowered and thus the power density is decreased [56].

## 4. Conclusions

The solid polymer electrolyte systems based on a Dex-HEC blend and doped with ammonium bromide (NH_4_Br) were effectively prepared viathe solution casting technique. The 40 wt% Dex-60 wt% HEC blend has the most suitable polymer host ratio. A 20 wt% NH_4_Br increased the conductivity to up to (1.47 ± 0.12) × 10^−4^ S cm^−1^. The number density and mobility of ions in the highest conducting electrolyte (SL4) were 1.58 × 10^21^ cm^−3^ and 6.27 × 10^−7^ V^−1^s^−1^ cm^2^, respectively. Up to 1.7V, the electrolyte is potentially stable. The EDLC’s specific capacitance and energy density were stable at 13.1 F g^−1^ and 1.31 Wh kg^−1^, respectively. Even though the EDLC has been subjected to rapid charge-discharge, there are no extreme changes in the internal resistance value. The pattern of power density is highly related to the ESR, where it becomes constant at 109 W kg^−1^. The performance of the EDLC at a cycle below 450 is unstable because ions in the electrolyte are still in the process of recognizing the pattern of adsorption/desorption and conduction. Thus, it can be concluded that the Dex-HEC-NH_4_Br-based EDLC has shown an excellent performance. A plasticizer or filler can be added to the electrolyte to enhance the ionic conductivity for future improvement. The procedure and materials used in electrode fabrication can be modified to enhance the EDLC’s performance.

## Figures and Tables

**Table 1:** Designation for the polymer blend systems and their respective degree of crystallinity.

Dextran:HEC Composition (wt%)	Designation
0:100	BL0
10:90	BL1
20:80	BL2
30:70	BL3
40:60	BL4
50:50	BL5
60:40	BL6
70:30	BL7
80:20	BL8
90:10	BL9
100:0	BL10

**Table 2:** Designation for the salted systems.

Dextran:HEC:NH_4_Br Composition (wt%)	Designation
40:60:0	BL4
38:57:5	SL1
36:54:10	SL2
34:51:15	SL3
32:48:20	SL4
30:45:25	SL5
28:42:30	SL6

**Table 3:** Designation for the polymer blend systems and their respective degree of crystallinity.

Designation	Degree of Crystallinity (*χ_c_*)
BL0	30.53
BL1	29.97
BL2	28.57
BL3	24.44
BL4	20.17
BL5	24.59
BL6	28.52
BL7	29.02
BL8	29.93
BL9	30.88
BL10	32.06

**Table 4:** The value of *E_ac_* for the salted electrolytes.

Electrolyte	*E_ac_*(eV)
SL1	0.68
SL2	0.65
SL3	0.54
SL4	0.26
SL5	0.38
SL6	0.61

**Table 5:** Room temperature circuit elements for the salted electrolytes.

Electrolyte	*k*_1_ (F^−1^)	*k*_2_ (F^−1^)	*C*_1_ (F)	*C*_2_ (F)
**SL1**	8.00 × 10^8^	3.70 × 10^5^	1.25 × 10^−9^	2.70 × 10^−6^
**SL2**	1.40 × 10^8^	3.68 × 10^5^	7.14 × 10^−9^	2.72 × 10^−6^
**SL3**	1.20 × 10^8^	3.65 × 10^5^	8.33 × 10^−9^	2.74 × 10^−6^
**SL4**	-	3.80 × 10^4^	-	2.63 × 10^−5^
**SL5**	-	9.50 × 10^4^	-	1.05 × 10^−5^
**SL6**	-	1.02 × 10^5^	-	9.80 × 10^−6^

**Table 6:** High temperature circuit elements for the SL4 electrolyte.

Temperature (K)	*k* (F^−1^)	*C* (F)
**298**	3.80 × 10^4^	2.63 × 10^−5^
**303**	3.55 × 10^4^	2.82 × 10^−5^
**313**	2.92 × 10^4^	3.42 × 10^−5^
**323**	2.10 × 10^4^	4.76 × 10^−5^
**333**	1.45 × 10^4^	6.90 × 10^−5^
**343**	1.10 × 10^4^	9.09 × 10^−5^

**Table 7:** Capacitance values from cyclic voltammetry (CV) against scan rates.

Scan Rates (mV s^−1^)	Capacitance (F g^−1^)
100	9.51
50	13.41
20	20.84
10	29.70
5	36.69

**Table 8:** Various reported activated carbon-based EDLC studies with their respective value of specific capacitance.

Polymer Electrolyte	Current Density	*C_CD_* (F/g)	Cycles	Reference
Dex-NH_4_Br	0.063 mA/cm^2^	2.05	100	[52]
HEC-MgTf_2_-EMIMTf-SiO_2_	0.400 A/g	25.00	1600	[63]
CS-MC-NH_4_I-Gly	0.400 mA/cm^2^	9.70	100	[64]
PVA-Dex-NH_4_I	0.500 mA/cm^2^	4.20	100	[53]
PVA-LiClO_4_-TiO_2_	-	12.50	1000	[65]
PVA-Naft-BmImBr	0.200 A/g	16.32	1000	[66]
PS-MC-NH_4_NO_3_-Gly	0.200 mA/cm^2^	31.00	1000	[67]
Dex-HEC-NH_4_Br	0.250 mA/cm^2^	31.70	8000	This work

## Data Availability

Data is contained within the article.
